# Myo-inositol oxygenase is important for the removal of excess myo-inositol from syncytia induced by *Heterodera schachtii* in Arabidopsis roots

**DOI:** 10.1111/nph.12535

**Published:** 2013-10-01

**Authors:** Shahid Siddique, Stefanie Endres, Miroslaw Sobczak, Zoran S Radakovic, Lena Fragner, Florian M W Grundler, Wolfram Weckwerth, Raimund Tenhaken, Holger Bohlmann

**Affiliations:** 1 Division of Plant Protection, Department of Crop Sciences, University of Natural Resources and Life SciencesA-1019, Vienna, Austria; 2 Plant Physiology, University of SalzburgHellbrunnerstr. 34, A-5020, Salzburg, Austria; 3 Department of Botany, Warsaw University of Life Sciences (SGGW)02-787, Warsaw, Poland; 4 INRES, Department of Molecular Phytomedicine, University of BonnKarlrobert–Kreiten–Str. 13, 53115, Bonn, Germany; 5 Department of Molecular Systems Biology, University of ViennaA-1090, Vienna, Austria

**Keywords:** Arabidopsis, ascorbic acid, galactinol, *Heterodera schachtii*, myo-inositol oxygenase, roots, syncytium

## Abstract

- The enzyme myo-inositol oxygenase is the key enzyme of a pathway leading from myo-inositol to UDP-glucuronic acid. In Arabidopsis, myo-inositol oxygenase is encoded by four genes. All genes are strongly expressed in syncytia induced by the beet cyst nematode *Heterodera schachtii* in Arabidopsis roots. Here, we studied the effect of a quadruple myo-inositol oxygenase mutant on nematode development.
- We performed metabolite profiling of syncytia induced in roots of the myo-inositol oxygenase quadruple mutant. The role of galactinol in syncytia was studied using Arabidopsis lines with elevated galactinol levels and by supplying galactinol to wild-type seedlings.
- The quadruple myo-inositol oxygenase mutant showed a significant reduction in susceptibility to *H. schachtii*, and syncytia had elevated myo-inositol and galactinol levels and an elevated expression level of the antimicrobial thionin gene *Thi2.1*. This reduction in susceptibility could also be achieved by exogenous application of galactinol to wild-type seedlings.
- The primary function of myo-inositol oxygenase for syncytium development is probably not the production of UDP-glucuronic acid as a precursor for cell wall polysaccharides, but the reduction of myo-inositol levels and thereby a reduction in the galactinol level to avoid the induction of defence-related genes.

## Introduction

Plants are continuously attacked by a variety of pathogens and pests. One group that preferentially attacks the roots is the plant parasitic nematodes. They have developed life styles that make them important pests for many cultivated plants. Migratory nematodes browse the root surface or cortex to parasitize single root cells, whereas sessile nematodes retrieve nutrients only from feeding sites that they induce in the roots of their host plants. Nematode infections can severely reduce the yield of crop plants, and their economic impact has been estimated at $157 billion yr^−1^ (Abad *et al*., 2008). Root-knot nematodes, especially the genus *Meloidogyne*, induce several giant cells from which they feed alternately. Their name is derived from the galls that form around their feeding sites through enhanced division of root cells. Cyst-forming nematodes are the second most economically important group of sessile plant parasitic nematodes, which induce a feeding site that is a syncytium. The female nematodes produce several hundred eggs inside their body, which thereby enlarges to a lemon shape and hardens to form a cyst when the female dies. The eggs can survive in the cyst for many years, making these nematodes difficult to eradicate. The infective juveniles (J2) hatch from the eggs under favourable conditions and infect the roots of host plants.

The development of the syncytium starts from a single root cell inside the vascular cylinder, which is selected by the J2 and pierced with its stylet. It is commonly agreed that the nematode injects proteins produced in its oesophageal glands into the root cell to induce the development of the syncytium, although the nature of these secretions is still largely unknown (Hewezi & Baum, 2013). From this initial syncytial cell the syncytium develops through local cell wall dissolution of neighbouring cells (Wyss & Grundler, 1992). In its final size, a syncytium associated with female nematodes consists of several hundred root cells whose nuclei enlarge through endoreduplication. Furthermore, the ultrastructure of the syncytial elements shows drastic changes as the large central vacuole is replaced by several small vacuoles and increasing cytoplasm containing large numbers of ribosomes and mitochondria (Sobczak *et al*., 1999). The ultrastructure of syncytia implicates a high metabolic activity, which is necessary to fulfil the needs of the developing nematode, which continuously withdraws nutrients from its feeding site. During the course of these processes, the osmotic pressure of syncytia increases to exceed that of adjacent cells by several fold.

The incorporation of root cells into syncytia requires the dissolution of cell walls, and it has been shown that a number of plant proteins are involved in this process, including expansins, cellulases and pectinases. The expression of these proteins must be tightly regulated, and some of the genes encoding expansins and cell wall-degrading enzymes are specifically induced in syncytia or in the surrounding tissue (Wieczorek *et al*., 2006, 2008; Szakasits *et al*., 2009). In addition to processes that degrade cell walls, the development of syncytia also requires the synthesis of new cell wall materials. The outer cell wall of syncytia is thickened to withstand the high osmotic pressure inside syncytia (Golinowski *et al*., 1996). In addition, cell walls of syncytia that are associated with female nematodes have been shown to develop pronounced cell wall ingrowths at the interface with xylem vessels (Siddique *et al*., 2012). Similar cell wall ingrowths have been found in the transfer cells of plants (Jones & Northcot, 1972). It has been proposed that their function is to increase the surface, thus allowing a higher exchange of solutions.

In Arabidopsis, the major precursor of cell wall polysaccharides is UDP-glucuronic acid, which can be produced through two different pathways. Under normal growth conditions, the enzyme UDP-glucuronic acid dehydrogenase (UGD) supplies the majority of UDP-glucuronic acid from UDP-glucose. A second pathway involves myo-inositol oxygenase (MIOX), which converts myo-inositol to d-glucuronic acid, which is thereafter converted into d-glucuronic acid-1-phosphate and, finally, into UDP-glucuronic acid, catalysed by glucuronokinase and UDP-sugar pyrophosphorylase (USP), respectively (Supporting Information Fig. S1).

Myo-inositol is produced from glucose-6-phosphate through the rate-limiting conversion to myo-inositol-3-phosphate catalysed by myo-inositol-1-phosphate synthase (MIPS), followed by dephosphorylation to myo-inositol by myo-inositol monophosphatases (IMP; Loewus & Loewus, 1982). In addition to being converted to UDP-glucuronic acid, myo-inositol serves as a precursor for phytic acid, phosphatidylinositol phosphate, myo-inositol phosphates and sphingolipids, which have been implicated in a variety of cellular processes (Irvine & Schell, 2001; Tan *et al*., 2007). Furthermore, it has been shown that myo-inositol is important for embryo development as a precursor for phosphatidylinositol and phosphatidylinositides, which are essential for auxin-regulated embryogenesis (Luo *et al*., 2011). Arabidopsis contains small gene families with three genes each for MIPS and IMP. *MIPS1* is expressed in most Arabidopsis tissues and developmental stages, whereas *MIPS2* and *MIPS3* have been found to be especially expressed in vascular or related tissues (Donahue *et al*., 2010).

Myo-inositol is also a precursor for galactinol through the coupling with UDP-galactose, which is catalysed by galactinol synthase (GS). Galactinol can be further reacted with sucrose to produce raffinose; this reaction recycles myo-inositol. Although there are 10 GS genes in the Arabidopsis genome, there is only one gene coding for raffinose synthase (RS). Galactinol and raffinose have been proposed as osmoprotectants in plants. In line with this, several GS genes are induced by abiotic stresses, and the overexpression of GS increases the drought resistance of transgenic plants (Taji *et al*., 2002). However, the Arabidopsis RS mutant, which is unable to produce raffinose, but accumulates higher levels of galactinol, did not show any difference in cold acclimation or freezing tolerance, indicating that raffinose is not involved, but that galactinol might play a role (Zuther *et al*., 2004).

Arabidopsis possesses a small gene family of four genes encoding UGD (*UGD1*, *UGD2*, *UGD3* and *UGD4*; Klinghammer & Tenhaken, 2007). *UGD1* is weakly expressed in roots, whereas the other three genes (*UGD2*, *UGD3* and *UGD4*) are strongly expressed in roots. We have recently studied the expression of these genes in syncytia using promoter::GUS lines (Siddique *et al*., 2012). All four genes were expressed in syncytia, *UGD2* and *UGD3* as early as 1 d post-inoculation (dpi), whereas the expression of *UGD1* and *UGD4* was detected starting at 2 dpi. A mutant analysis revealed that the single mutants Δ*ugd2* and Δ*ugd3* support the development of fewer and smaller females and smaller syncytia when compared with wild-type plants. The double mutant ΔΔ*ugd23* showed an even stronger effect than the single mutants. The ultrastructure of syncytia in the ΔΔ*ugd23* double mutant revealed an electron-translucent cytoplasm with degenerated cellular organelles and an absence of cell wall ingrowths in syncytia associated with female nematodes. Thus, *UGD2* and *UGD3* are needed for cell wall ingrowth formation in syncytia (Siddique *et al*., 2012).

Four genes in Arabidopsis encode MIOX. *MIOX1* and *MIOX2* are expressed preferentially in seedlings, whereas *MIOX4* and *MIOX5* are highly expressed in pollen (Kanter *et al*., 2005). A quadruple (*miox1/2/4/5*) mutant that incorporates T-DNA insertions in all four *MIOX* genes has been described (Endres & Tenhaken, 2011). This mutant showed a severe reduction in transcripts for all four *MIOX* genes. However, except for *MIOX2*, transcripts for the other three *MIOX* genes could still be detected at a level of 2–14% of their abundance in the wild-type. The *miox1/2/4/5* mutant did not show any visible phenotype and produced viable pollen. However, it was found that the incorporation of myo-inositol-derived sugars into cell walls was strongly (> 90%) inhibited. All four *MIOX* genes are expressed at high levels in syncytia (Siddique *et al*., 2009). Double mutants of the four *MIOX* genes showed a significantly reduced development of *H. schachtii*, indicating the importance of the MIOX pathway for the development of syncytia. We therefore suspected that this might be caused by impairment in cell wall biosynthesis; however, we could not detect differences in the cell wall composition of *miox* double mutants or at the ultrastructural level. Here, we have extended this work using the quadruple mutant. We provide evidence that the importance of MIOX in syncytium development is not the production of cell wall precursors, but rather the removal of excess myo-inositol from syncytia.

## Materials and Methods

### Plant cultivation

*Arabidopsis thaliana* (L.) Heynh plants were surface sterilized for 10 min in 6% (w/v) sodium hypochlorite and subsequently washed several times with sterile water. Plants for infection with nematodes were grown on Knop medium (0.2%) in Petri dishes (9 cm) as described by Sijmons *et al*. (1991) in a growth chamber at 25°C in a 16 h : 8 h light : dark cycle.

### Nematode infection assays

*Heterodera schachtii* Schmidt cysts were harvested from *in vitro* stock cultures on mustard (*Sinapis alba* cv Albatros) roots growing on Knop medium (0.2%). The addition of 3 mM ZnCl_2_ stimulated the hatching of the juveniles. Three to 4 d later, J2s were collected and resuspended in 0.7% (w/v) gelrite (Duchefa, Haarlem, the Netherlands). Twelve-day-old Arabidopsis plants were inoculated with 60–70 J2 *H. schachtii* juveniles under sterile conditions. Ten plants were used in one Petri dish and experiments were repeated three times with 40–50 plants in one replicate. The numbers of males and females per plant were counted at 14 dpi and the data were analysed by *t*-test (*P *<* *0.05) or single-factor ANOVA (*P *<* *0.05). In the case of ANOVA, if the *F*-statistic was higher than *F*-critical, Fisher′s least-significant difference (LSD) test was applied.

### Syncytium and female size measurement

The sizes of syncytia and associated female nematodes were measured at 10 dpi. For each line, 50 syncytia associated with females were randomly selected and photographed by an Axiovert 200M (Zeiss AG, Germany) using a Zeiss Axiocam digital camera. The syncytia and females were outlined using the Axioversion Kontour tool. The individual measurements were used to calculate the average size of syncytium and female. Data were further statistically analysed using single-factor ANOVA (*P *<* *0.05) and Fisher′s LSD test.

### RNA isolation

Root sections containing syncytia were cut and immediately frozen in liquid nitrogen. Corresponding control sections from uninfected plants were cut as controls. Three biological replicates were performed for both root and syncytium. Total RNA was isolated using an RNeasy Plant Mini Kit (Qiagen, Hilden, Germany) according to the manufacturer′s instructions, including DNaseI (Qiagen) digestion. The quality and quantity of RNA were assessed using an Agilent 2100 bioanalyser according to the manufacturer′s instructions (Agilent Technologies, Palo Alto, CA, USA). Reverse transcription was performed with a SuperScript III reverse transcriptase (Invitrogen, Carlsbad, CA, USA) and random primers (oligo(dN)6) according to the manufacturer′s instructions to prepare first-strand cDNA.

### Quantitative reverse transcription-polymerase chain reaction (RT-PCR) of gene expression in syncytia

Quantitative real-time RT-PCR (qPCR) of *UGD* gene expression in syncytia collected from *miox1/2/4/5* mutants and wild-type plants was performed with an ABI PRISM 7300 Sequence Detector (Applied BioSystems, Foster City, CA, USA). Primer sequences can be found in Table S1. Each qPCR sample contained 12.5 μl of Platinum SYBR Green qPCR SuperMix (Invitrogen) with UDG and 6-Carboxyl-X-Rhodamine (ROX), 2 mM of MgCl_2_, 0.5 μl of forward and reverse primer (10 μM), 2 μl of cDNA and water to reach a total reaction volume of 25 μl. All samples were analysed in three biological and three technical replicates. Control reactions with no cDNA template ruled out false positives, and melting curve analysis was performed to assess the possible primer dimer formation. The 18S rRNA gene was used as an internal reference as described previously (Hofmann & Grundler, 2007). Results were obtained using the Sequence Detection Software SDS v2.0 (Applied BioSystems).

### Ultrastructure analysis

Arabidopsis plants were grown and inoculated with *H. schachtii* as described earlier. Root and syncytial sections from wild-type and *miox1/2/4/5* mutant plants were collected and processed at 5, 10 and 15 dpi, as described previously (Siddique *et al*., 2012).

### Myo-inositol measurements

Syncytial and uninoculated root samples were collected as described before for RNA isolation. The myo-inositol content of the samples was measured as described by Endres & Tenhaken (2011). Briefly, samples were homogenized and extracted with a mixture of methanol, chloroform and water (10 : 4 : 4, v/v), as described by Fiehn *et al*. (2000). They were incubated at 70°C for 20 min and afterwards centrifuged at 18 000 ***g*** for 3 min; the supernatant was transferred to a fresh Eppendorf tube and a mixture of water and chloroform was added (1 :** **5.75, v/v). After incubation for 5 min at 37°C, samples were centrifuged at 7000 ***g*** for 15 min. Supernatants containing soluble sugars were dried in a SpeedVac centrifuge and resuspended in distilled water. These resuspended samples were analysed on an ICS3000 system (Dionex, Vienna, Austria) using a CarboPac MA1 analytical column (Dionex) for inositol measurements, as described previously (Endres & Tenhaken, 2011).

### Metabolome analysis of syncytium

At 10 dpi, root and syncytial segments associated with female nematodes were collected after removal of the nematodes and immediately frozen in liquid nitrogen as described above. Samples were homogenized and soluble metabolites were extracted by methanol/chloroform extraction (Weckwerth *et al*., 2004). Samples were dried under vacuum for further processing. Chemical derivatization, including methoxyamination and trimethylsilylation, of dried samples was performed as described by Mari *et al*. (2012). One microlitre of derivatized sample was used to perform gas chromatography-mass spectrometry (GC-MS) analysis employing a Thermofisher gas chromatograph coupled to a Triple Quadrupole mass analyser (Thermo Scientific TSQ Quantum GC™, Bremen, Germany). Metabolite derivatives were identified and annotated using retention time information and by matching the mass spectrum to an in-house mass spectral library. The areas of the peaks were calculated and normalized to the fresh weight of the samples. All samples were measured in four biological replicates. Normalized responses were further used for statistical analysis. For statistical analysis, further normalization, means, standard deviations and *t*-tests (*P *<* *0.05) were performed using the software MultiExperiment Viewer Version 4.4.0 (http://www.tm4.org). For the generation of a volcano plot, the values were transformed into log_2_ scale.

## Results

### Infection assay for *miox1/2/4/5*

**Figure 1:**
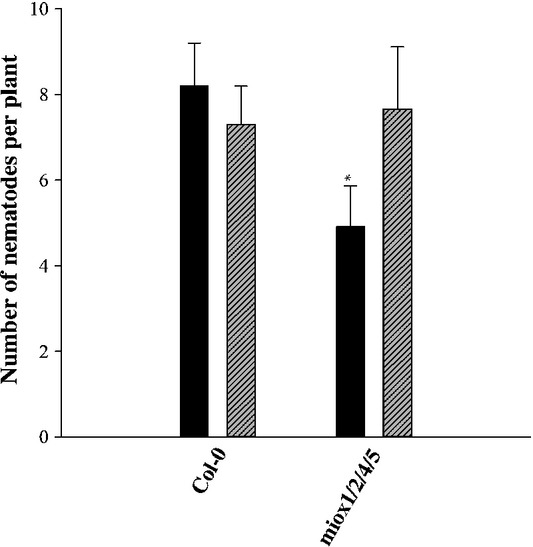
Infection assay with *Heterodera schachtii* of quadruple *miox* mutants (*miox1/2/4/5*) compared with wild-type Arabidopsis plants. Numbers of males and females were counted at 14 d post-inoculation (dpi). Columns represent the average number of males (hatched bars) and females (closed bars). Significant differences: *, *P *<* *0.05; *t*-test. The statistical significance was determined by three independent replicates. Values are means ± SE, *n* = 3.

We have recently described *miox* double mutants and found that they were less susceptible to infection by *H. schachtii* (Siddique *et al*., 2009). In the meantime, the quadruple (*miox1/2/4/5*) *miox* mutant, which incorporates T-DNA insertions in all four *MIOX* genes, has become available (Endres & Tenhaken, 2011). We grew this mutant on Knop medium as described in the Materials and Methods section, and we did not observe any change in plant growth and development under these conditions. Plants were inoculated with *H. schachtii* J2 juveniles after 12 d. At 10 dpi, we cut root segments harbouring syncytia and extracted RNA to compare the expression of the four *MIOX* genes in wild-type plants and the *miox1/2/4/5* mutant. Our analysis confirmed previous findings, as we did not detect any signal for *MIOX2* in *miox1/2/4/5*. In addition, the levels of the remaining three *MIOX* genes were severely reduced in *miox1/2/4/5* when compared with wild-type plants (Table S2). We counted the numbers of males and females after 2 wk of inoculation, and we hypothesized that the *miox1/2/4/5* mutant would be less susceptible to nematodes than the double mutants (*miox1 + 2*; *miox4 + 5*), which have been found to show a reduction in the number of females of *c*. 40% (Siddique *et al*., 2009). However, the *miox1/2/4/5* plants showed a similar reduction in the number of females to the *miox* double mutants (*miox1 + 2*; *miox4 + 5*; Fig.1; fig. 6 in Siddique *et al*., 2009).

**Figure 2:**
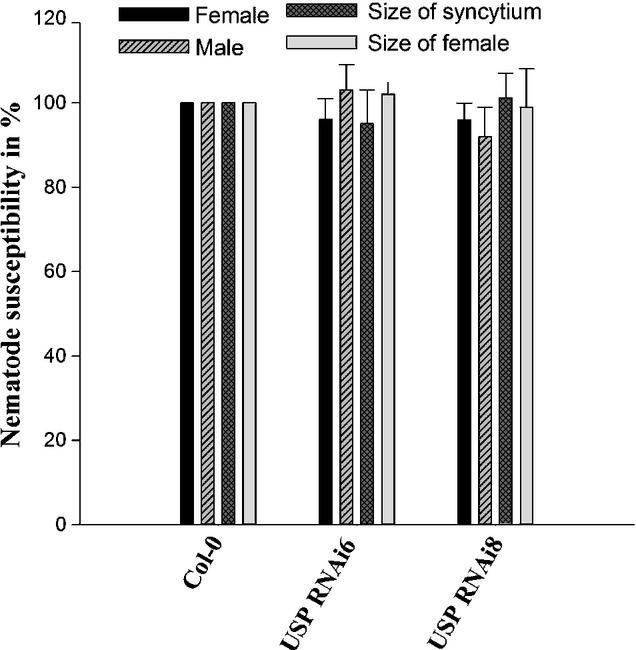
Infection assay with *Heterodera schachtii* of *USP* RNAi lines (Kotake *et al*., 2007) compared with wild-type Arabidopsis plants. Numbers of males and females were counted at 14 d post-inoculation (dpi). Columns represent the average numbers of males and females, and sizes of females and syncytia. *P *<* *0.05; *t*-test. The statistical significance was determined by three independent replicates. Values are means ± SE, *n* = 3.

The MIOX pathway leads from myo-inositol to UDP-glucuronic acid. In addition to MIOX, the enzymes glucuronokinase and USP are involved in this pathway (Fig. S1). Therefore, it would have been interesting to test mutants which lack these enzymes, but no mutant has been identified for the glucuronokinase and the available USP mutant is pollen sterile (Schnurr *et al*., 2006; Kotake *et al*., 2007). However, *USP* RNAi plants were available (Kotake *et al*., 2007) which had been produced by expressing an antisense construct of the *USP* cDNA. Six lines were tested by these authors for UDP-l-arabinose pyrophosphorylase activity and two of the lines (6 and 8) showed a reduction in activity to 25% of wild-type plants. We tested these two antisense lines against *H. schachtii*, but our analysis did not show any change in susceptibility of these plants to *H. schachtii* (Fig.2).

### Ultrastructure of syncytia

Previously, we analysed the sugar composition and ultrastructure of cell walls from syncytia developing in *miox* double mutants, but found no differences in comparison with wild-type plants (Siddique *et al*., 2009). With the availability of the *miox1/2/4/5* mutant, we studied the development of syncytia at the ultrastructural level in a time course analysis at 5, 10 and 15 dpi. As the MIOX pathway leads to the synthesis of a central precursor for cell wall polysaccharides, we expected that cell walls of syncytia developing in *miox1/2/4/5* roots might show some differences when compared with syncytia developing in wild-type plants. However, ultrastructural analysis did not reveal any significant change in the roots or syncytia induced in *miox1/2/4/5* plants when compared with wild-type plants (Fig. S2).

### Expression of *UGD* genes

**Figure 3:**
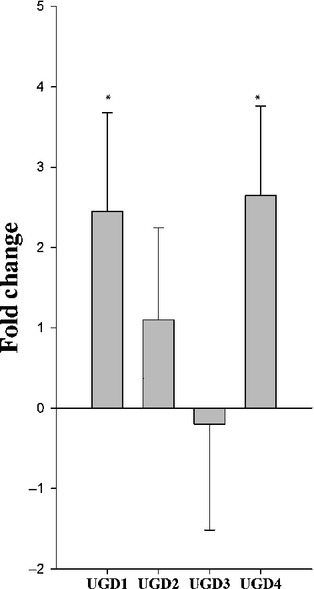
Expression of *UDP-glucuronic acid dehydrogenase* (*UGD*) genes in syncytia induced by *Heterodera schachtii* in the Arabidopsis *miox* quadruple mutant. Transcripts of *UGD1*, *UGD2*, *UGD3* and *UGD4* were measured by quantitative real-time reverse transcription-polymerase chain reaction (qPCR) at 10 d post-inoculation (dpi). Values are means ± SE, *n* = 3. Significant differences: *, *P *<* *0.05; ANOVA and least-significant difference (LSD).

The fact that we did not see any change in the ultrastructure of syncytial cell walls suggested that there was probably a sufficient level of UDP-glucuronic acid (UDP-GlcA) as a precursor for cell wall polysaccharides. We therefore measured the expression of the *UGD* gene family (*UGD1*, *UGD2*, *UGD3* and *UGD4*) in syncytia developing on *miox1/2/4/5* and wild-type plants by qPCR. UGD is the key enzyme acting in the alternative pathway for the synthesis of UDP-GlcA. Of the four *UGD* genes, *UGD1* and *UGD4* were upregulated in *miox1/2/4/5* syncytia when compared with wild-type plants (Fig.3). This upregulation of two *UGD* genes (*UGD1* and *UGD4*) is correlated with the lack of a syncytium-specific cell wall phenotype.

### Syncytia have a low myo-inositol content

**Figure 4:**
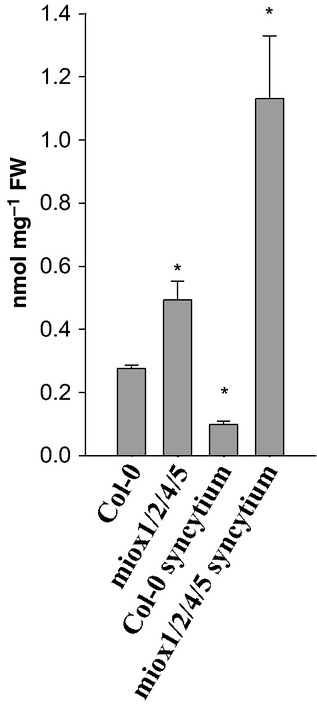
High-performance liquid chromatography (HPLC) analysis of myo-inositol levels in Arabidopsis roots and syncytia induced by *Heterodera schachtii*. Myo-inositol levels of wild-type and *miox1/2/4/5 *Arabidopsis mutants were measured at 10 d post-inoculation (dpi). Peak identity was verified with authentic standards. Values are means ± SE, *n* = 3. Significant differences: *, *P *<* *0.05; ANOVA and least-significant difference (LSD).

Recently, it has been shown that MIOX controls the level of myo-inositol in Arabidopsis plants (Endres & Tenhaken, 2009). As all four *MIOX* genes are strongly expressed in syncytia, their myo-inositol content should be rather low. We measured the level of myo-inositol in syncytia and in uninfected roots at 10 dpi (Fig.4). Our analysis showed that there was indeed a significant reduction in the myo-inositol level in syncytia developing in wild-type plants when compared with uninfected roots. However, the downregulation of the MIOX pathway in the *miox1/2/4/5* mutant resulted in the accumulation of myo-inositol in syncytia. Similarly, the difference between uninfected *miox1/2/4/5* and wild-type roots was also significant (Fig.4).

### Ascorbic acid (AsA) measurement

**Figure 5:**
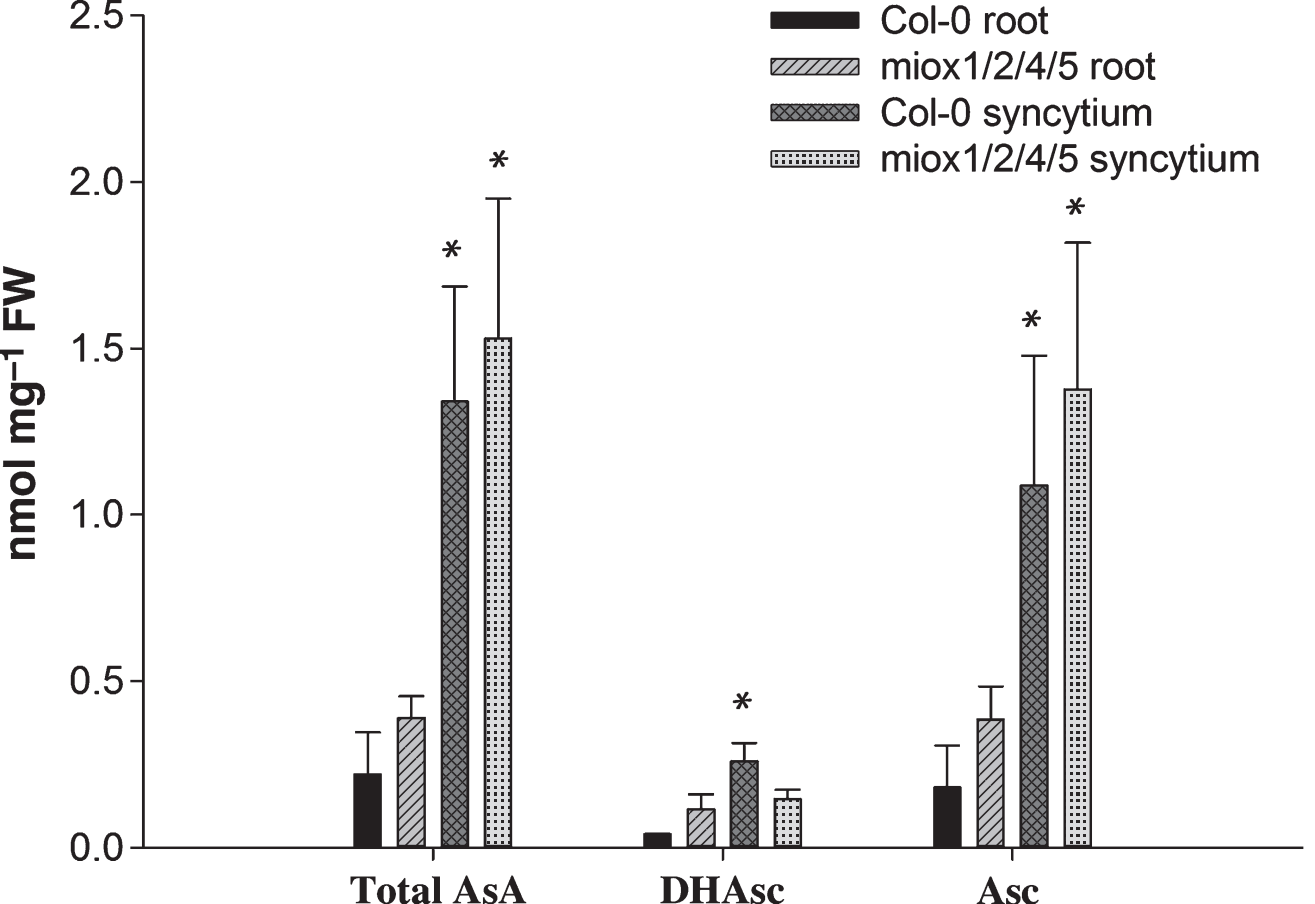
High-performance liquid chromatography (HPLC) analysis of ascorbate levels in uninfected roots and syncytia induced by *Heterodera schachtii* in wild-type Arabidopsis plants and *miox1/2/4/5* mutants at 10 d post-inoculation (dpi). Total AsA, total ascorbic acid (Asc + DHAsc); DHAsc, dehydroascorbic acid; Asc, ascorbic acid. Peak identity was verified with authentic standards. Values are means ± SE, *n* = 3. Significant differences: *, *P *<* *0.05; ANOVA and least-significant difference (LSD).

Previous studies have claimed a role for the MIOX pathway in the synthesis of AsA (Lorence *et al*., 2004; Zhang *et al*., 2008). In order to test whether the change in susceptibility of *miox1/2/4/5* plants was caused by a change in the level of AsA, we measured AsA in uninfected roots and syncytia (Fig.5). There was a significant increase in the amount of total AsA in syncytia when compared with control roots in wild-type plants. However, the downregulation of the MIOX pathway did not cause any significant change in the AsA levels in roots or in syncytia induced in both lines. These results indicate that the *miox1/2/4/5* mutants are not significantly altered in AsA synthesis in roots and syncytia.

### Metabolite profiling

**Figure 6:**
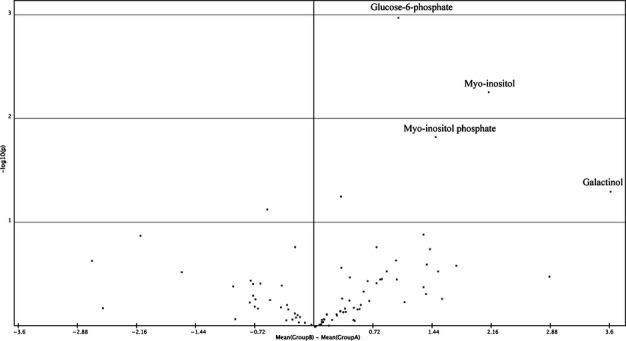
Comparison of wild-type syncytia and *miox1/2/4/5* syncytia induced by *Heterodera schachtii* in Arabidopsis roots by gas chromatography-mass spectrometry (GC-MS)-based metabolite profiling. The figure shows a volcano plot (www.tm4.org) with the *x*-axis representing the difference between the mean of *miox1/2/4/5* (GroupB) and wild-type (GroupA) syncytia on a log_2_ scale. The *y*-axis is –log_10_ of the *P*-value (*P*-value, 0.05) from four to five biological replicates. Red dots represent significantly different metabolites.

At this point, our results did not indicate why the downregulation of *MIOX* expression in syncytia led to a reduced susceptibility to *H. schachtii*. We therefore took a non-targeted approach and performed metabolite profiling by GC-MS to compare *miox1/2/4/5* and wild-type syncytia. Root segments containing syncytia associated with female nematodes were collected at 10 dpi in four to five biological replicates. Our analysis showed that there were four metabolites which accumulated significantly in *miox1/2/4/5* syncytia when compared with syncytia formed in wild-type roots (Fig.6, Table S3). These included galactinol, myo-inositol, myo-inositol phosphate and glucose-6-phosphate. This analysis confirmed our previous finding that myo-inositol accumulated in syncytia induced in *miox1/2/4/5* roots. Myo-inositol phosphate is a precursor of myo-inositol and glucose-6-phosphate is a common metabolite of primary metabolism, as well as a precursor of myo-inositol phosphate. Galactinol is produced from myo-inositol and UDP-galactose and can be further converted to raffinose (Fig. S1). It has been proposed to be involved in the resistance responses of plants (Kim *et al*., 2008; Cho *et al*., 2010). This indicated that the reduced susceptibility of *miox* mutants to *H. schachtii* might be caused by the increased galactinol level.

### Is the high galactinol content responsible for the reduced susceptibility of *miox* mutants?

**Figure 7:**
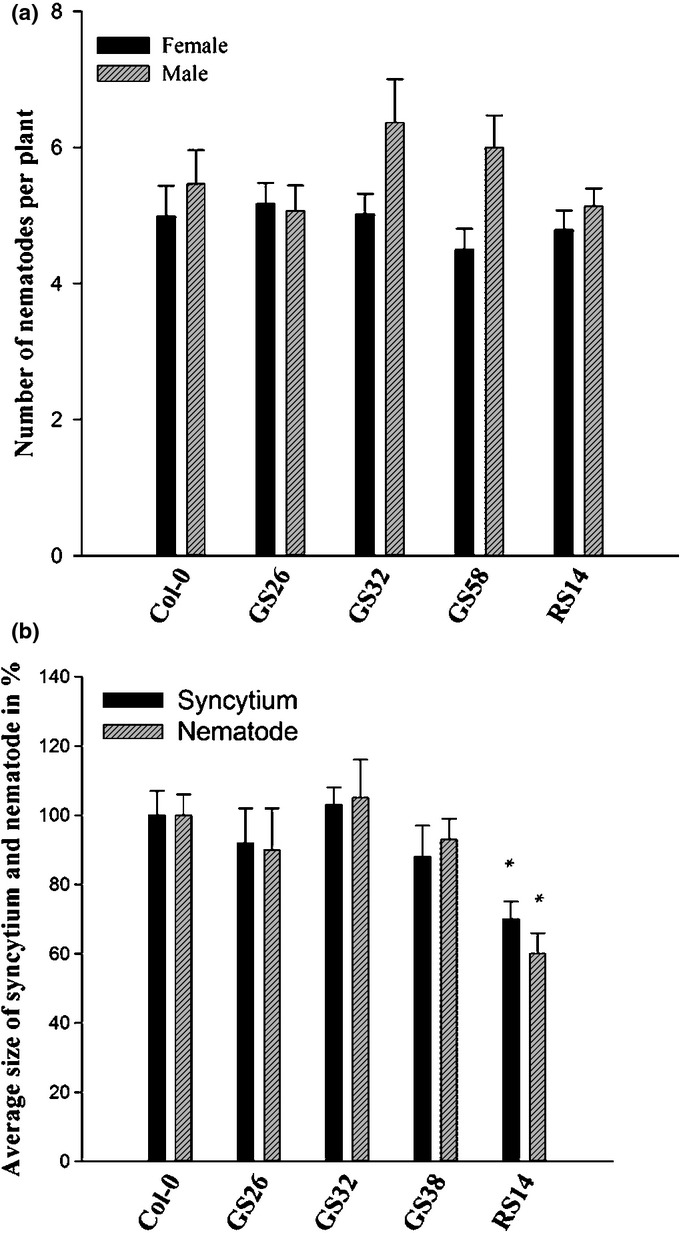
Infection assay with *Heterodera schachtii* of Arabidopsis galactinol synthase (GS)-overexpressing lines (GS26, GS32 and GS38) and the RS14 knockout mutant for raffinose synthase (RS14). Average numbers of males and females, and sizes of syncytia and females, were counted at 14 d post-inoculation (dpi). Significant differences from Col-0 wild-type plants on a log_2_ scale: *, *P* < 0.05; ANOVA and Fisher′s least-significant difference (LSD). The statistical significance was determined by three independent replicates. Values are means ± SE, *n* = 3.

In order to analyse whether the level of galactinol might influence the susceptibility of Arabidopsis plants to nematodes, we used the galactinol overexpression lines GS26, GS32 and GS58, and the RS mutant *RS14* (Zuther *et al*., 2004). GS26, GS32 and GS58 contain a GS cDNA from cucumber (*Cucumis sativus*), which was cloned into the binary vector pBin19 under the control of the cauliflower mosaic virus 35S promoter. RS14 is a knockout mutant for RS, which accumulates a relatively high level of galactinol as a result of blockage of the synthesis of raffinose. Plants were grown as described and then infected with juveniles of *H. schachtii*. Our analysis showed that the number of nematodes per plant was not changed considerably. However, the size of syncytia and females was reduced significantly in the RS14 mutant, but not in the GS overexpression lines (Fig.7). We reasoned that this difference between RS14 and the GS lines might be caused by the higher accumulation of galactinol in RS14 (five times) when compared with the GS lines (Zuther *et al*., 2004). However, *RS14* produces less raffinose; therefore, it was not clear whether the observed size effects were caused by a higher accumulation of galactinol or a reduced amount of raffinose.

**Figure 8:**
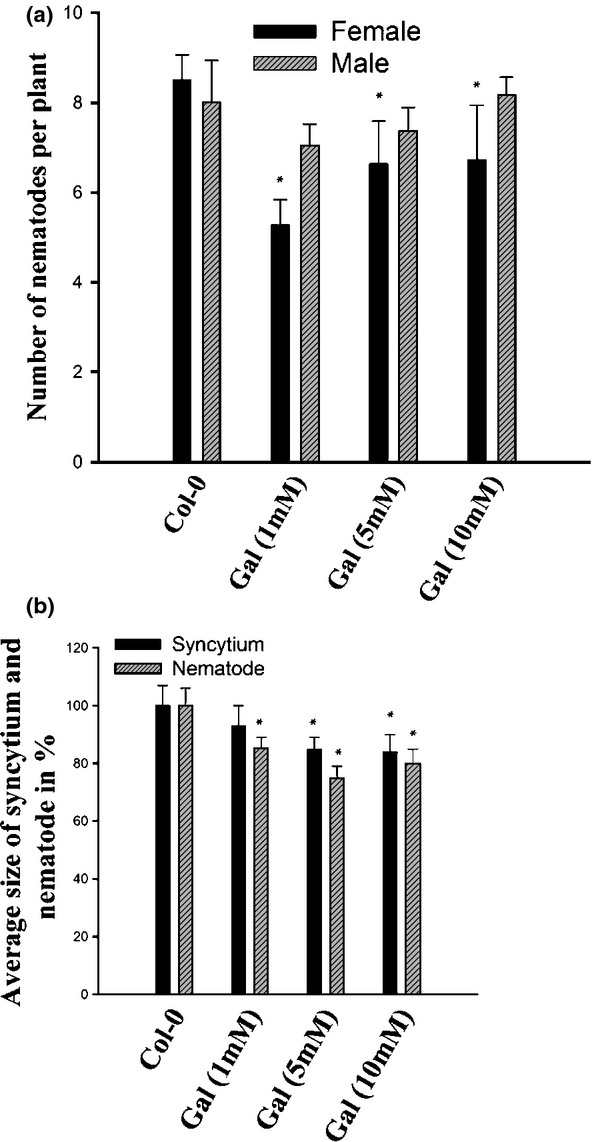
Infection assay with *Heterodera schachtii* using exogenous application of galactinol to Arabidopsis plants. Numbers of males and females, and sizes of syncytium and females, were counted at 14 d post-inoculation (dpi). Significant differences: *, *P *<* *0.05; ANOVA and least-significant difference (LSD). The statistical significance was determined by three independent replicates. Values are means ± SE, *n* = 3.

The results with the GS overexpression lines and the RS14 mutant indicated that a rather high level of galactinol was necessary to observe an effect on nematode development. Therefore, we applied galactinol exogenously in order to test its effect on nematode development. Our results showed a decrease in the number of females in plants treated with galactinol (Fig.8), but there was no statistically significant difference for 1, 5 and 10 mM galactinol. Similar to the number of nematodes, the size of syncytia and females decreased significantly on plates with exogenous galactinol. The only exception was 1 mM galactinol, which did not result in smaller syncytia.

### Expression of marker genes

**Figure 9:**
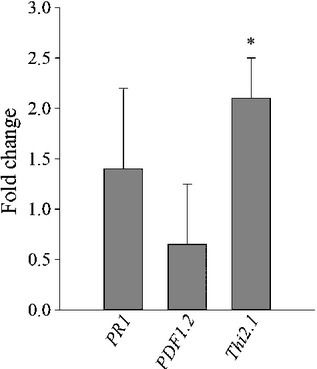
Expression of defence-related marker genes in Arabidopsis plants infected with *Heterodera schachtii*. Transcript levels of *PR1*, *PDF1.2* and *Thi2.1* were measured by quantitative real-time reverse transcription-polymerase chain reaction (qPCR) at 10 d post-inoculation (dpi) from syncytia of *miox1/2/4/5* mutants compared with the wild-type. Values are means ± SE, *n* = 3. Significant differences: *, *P *<* *0.05; ANOVA and least-significant difference (LSD).

If galactinol leads to reduced susceptibility to *H. schachtii*, this could be caused by an increase in the expression of defence-related genes, especially jasmonic acid (JA)-related genes (Cho *et al*., 2010). We therefore analysed the expression of the defence marker genes *PR1*, *PDF1.2* and *Thi2.1* by qPCR. *PR1* is a marker gene for salicylic acid (SA)-dependent gene expression (Uknes *et al*., 1992), whereas *PDF1.2* is a marker gene for JA/ethylene (Thomma *et al*., 1999). *Thi2.1* is regulated by JA (Epple *et al*., 1997; Bohlmann *et al*., 1998). RNA was extracted from 10-dpi syncytia developing in wild-type and *miox1/2/4/5* roots. Although there was no change in expression of *PR1* and *PDF1.2*, the expression of *Thi2.1* was upregulated significantly in *miox1/2/4/5* syncytia when compared with syncytia developing on wild-type roots (Fig.9).

## Discussion

The development of nematode-induced syncytia is accompanied by cell wall dissolution, but new cell wall polysaccharides are also required to enable syncytium expansion and full functionality. The major precursor of plant cell wall polysaccharides in plants is UDP-GlcA, which can be produced through two different pathways. Under normal growth conditions, the enzyme UGD supplies the majority of UDP-glucuronic acid from UDP-glucose. This hypothesis is also supported by our recent work in the context of plant–nematode interaction, which demonstrated that *UGD* genes are necessary for the formation of cell wall appositions in syncytia associated with female nematodes (Siddique *et al*., 2012). Alternatively, the MIOX biosynthetic pathway produces UDP-GlcA from myo-inositol. The Arabidopsis genome contains four *MIOX* genes and all four are strongly expressed in syncytia (Siddique *et al*., 2009), pointing to an important function for the MIOX pathway in syncytia. Indeed, we showed that double mutants with T-DNA insertions in two of the four *MIOX* genes were less susceptible to infection by *H. schachtii* (Siddique *et al*., 2012). However, the quadruple mutant, which has been tested in the present study, did not show a further decrease in susceptibility relative to the double mutants tested previously. As UDP-GlcA is an important precursor of several plant cell wall polysaccharides, we reasoned that the *miox* mutants might be affected by cell wall modifications. However, we could not detect differences in cell wall composition or ultrastructure of syncytia in the double mutants (Siddique *et al*., 2009) or quadruple mutant (Fig. S2). This was surprising, but we found that this could be explained by an upregulation of *UGD* genes in the quadrupule mutants. As UGD is involved in the alternative pathway for UDP-GlcA production as a precursor of cell wall polysaccharides, the upregulation of this pathway could at least partially rescue the *MIOX* quadruple mutant. It is also possible that residual expression of the three *MIOX* genes in *miox1/2/4/5*, together with the upregulation of two *UGD* genes, meets the minimal level of production of cell wall polysaccharides required for syncytium development. Nevertheless, the reduction in *MIOX* transcript levels in the double and quadruple mutants reduced significantly the susceptibility to *H. schachtii*. Therefore, the importance of the MIOX pathway does not seem to be a result of defects in cell wall synthesis. This raised the question of whether MIOX might be involved in other pathways important for syncytium development, other than the production of UDP-GlcA. This could be tested by knocking out the genes coding for glucuronokinase and/or USP, respectively, which act downstream of MIOX. Unfortunately, no mutants for glucuronokinase are available and the mutant for *USP* is pollen sterile and thus no homozygous mutants can be obtained. The available *USP* RNAi lines tested did not show any decrease in susceptibility, which might be caused by the fact that the USP enzyme activity level is only downregulated to *c*. 25% of the wild-type level (Kotake *et al*., 2007). All these results suggest that the reduced susceptibility of *miox* mutants is not caused by a decrease in cell wall polysaccharides. Another explanation for the reduced susceptibility of *miox* mutants might be a reduced level of AsA in syncytia. It has been postulated that the MIOX pathway is involved in the production of AsA (Lorence *et al*., 2004). However, our results showed that the level of AsA in the syncytia and roots of the *miox* quadruple mutant was not significantly different from that of wild-type tissues. This showed that the MIOX pathway was not involved in AsA production in roots and syncytia, and thus the change in susceptibility of the *MIOX* quadruple mutant was not caused by a reduced AsA level.

Thus far, therefore, our results could not explain the reduced susceptibility of *miox* mutants. Therefore, we took an unbiased approach and carried out metabolite profiling of syncytia developing in the roots of the quadruple mutant. The work reported here was performed with plants grown on Knop medium containing 1% sucrose (Sijmons *et al*., 1991), which is generally used for research on Arabidopsis–nematode interactions, because it allows the easy observation and extraction of syncytia. Not much is known about gene expression in syncytia from soil-grown Arabidopsis plants.

Compared with the wild-type, the content of myo-inositol was increased in the syncytia induced in mutant roots, as was also confirmed by high-performance liquid chromatography (HPLC). Similarly, the contents of myo-inositol phosphate (direct precursor of myo-inositol) and glucose-6-phosphate (a precursor for myo-inositol phosphate), were much higher in syncytia developing in the quadruple mutant.

### Is the high level of galactinol the reason for the reduced susceptibility of *miox* mutants?

Galactinol was another metabolite highly abundant in syncytia induced in the *miox* quadruple mutant. It is produced through the coupling of myo-inositol and UDP-galactose. A further reaction with sucrose produces raffinose and recycles myo-inositol. In the *miox* quadruple mutant, the high myo-inositol content favours the synthesis of galactinol, but did not result in an increase in the level of raffinose. Thus, it was possible that the high galactinol content in the *miox* mutants was the reason for the reduced susceptibility. We tested this possibility by increasing the content of galactinol independent of mutations in the MIOX pathway. Lines that overexpressed a *GS* gene from cucumber reached approximately two-fold higher galactinol levels, but did not show any statistically significant change in susceptibility to *H. schachtii*. However, a mutant of RS (*RS14*), with blockage in the final step leading to raffinose, supported smaller syncytia than did wild-type plants. Similarly, the female nematodes developing on these plants were also much smaller. The difference between the *RS14* mutant and the *GS*-overexpressing lines was that the RS14 mutant had an approximately four-fold higher galactinol level (Zuther *et al*., 2004), thus indicating that this high galactinol level might have been responsible for the observed effects. However, it could not be excluded with certainty that the reduced raffinose level in the RS14 mutant might have been the reason for the observed effects. We therefore treated the seedlings with 1, 5 and 10 mM galactinol to directly increase the galactinol level. This treatment also led to smaller syncytia and smaller female nematodes and to a decrease in the number of nematodes, which showed that the effect in the RS14 mutant was caused by the higher level of galactinol and not the reduced level of raffinose. There was no statistically significant difference between the three galactinol concentrations (with the exception of 1 mM which did not result in smaller syncytia). The explanation for this effect might be that only a limited amount of galactinol is taken up into syncytia. Recently, galactinol has been shown to be involved in resistance signalling in addition to its involvement in abiotic stress responses. A cucumber GS gene was found to be associated with priming induced by *Pseudomoas chlororaphis* O6 root colonization, leading to an increase in galactinol content. This effect could be copied by exogenous galactinol application (Kim *et al*., 2008). In Arabidopsis, one *GS* gene was specifically induced by *Botrytis cinerea* infection and by priming with *P. chlororaphis* O6 root colonization. This resistance was mediated through the JA pathway (Kim *et al*., 2008; Cho *et al*., 2010). Thus, galactinol might be a signalling compound for induced resistance, although it could not be excluded that the active compound might be a product of galactinol, such as raffinose. This view is supported by our results, showing that the expression of the *Thi2.1* gene was induced in syncytia formed in roots of the *miox* quadruple mutant. According to our transcriptome analysis (Szakasits *et al*., 2009), *Thi2.1* is not regulated in wild-type syncytia and roots. This gene codes for an antimicrobial thionin peptide and has been shown previously to be involved in the resistance against pathogens and is regulated through JA (Epple *et al*., 1997; Bohlmann *et al*., 1998). However, the JA/ethylene marker gene *PDF1.2* (Thomma *et al*., 1999) and the SA marker gene *PR1* (Uknes *et al*., 1992) were not upregulated in syncytia of the *miox* quadruple mutant, indicating that the galactinol effect in syncytia does not depend on the ethylene or SA pathways.

Further work is needed to discover which other genes might be upregulated by galactinol and might be involved in the reduced susceptibility of the *miox* quadruple mutant to *H. schachtii*. It is also possible that other defence-related genes are activated during the early stages of infection and syncytium development. Furthermore, using mutants of different resistance pathways should indicate whether galactinol acts only through the JA pathway in syncytia during plant–nematode interaction.

### Conclusion

In this work, we have analysed the importance of the MIOX pathway for the development of syncytia induced by *H. schachtii*. Our results showed that the downregulation of the MIOX pathway in syncytia led to an increased galactinol level. The increased galactinol level might be responsible for the reduced susceptibility of *miox* quadruple and double mutants, most probably via an upregulation of defence-related genes.

## Supporting Information

Fig. S1 Pathways for the synthesis of UDP-glucuronic acid, galactinol and derivatives. **Fig. S2** Electron microscope comparison of root and syncytia in Col-0 and miox quadruple mutants. **Table S1** Sequences of primers used in this work **Table S2** Expression of MIOX genes in syncytia of the miox quadruple mutant relative to Col-0 measured by quantitative real-time reverse transcription-polymerase chain reaction (qPCR) at 10 d post-inoculation (dpi) **Table S3** Comparison of wild-type syncytia and miox1/2/4/5 syncytia by gas chromatography-mass spectrometry (GC-MS)-based metabolite profiling
