# Supplementary material for: Myo-inositol oxygenase is important for the removal of excess myo-inositol from syncytia induced by Heterodera schachtii in Arabidopsis roots
Source: New Phytol. 2013 Oct 1;201(2):476–85. doi: 10.1111/nph.12535 (PMC4285123; doi:10.1111/nph.12535)
Supplement: Fig. S1 — Pathways for the synthesis of UDP-glucuronic acid, galactinol and derivatives. Fig. S2 Electron microscope comparison of root and syncytia in Col-0 and miox quadruple mutants. Table S1 Sequences of primers used in this work Table S2 Expression of MIOX genes in syncytia of the miox quadruple mutant relative to Col-0 measured by quantitative real-time reverse transcription-polymerase chain reaction (qPCR) at 10 d post-inoculation (dpi) Table S3 Comparison of wild-type syncytia and miox1/2/4/5 syncytia by gas chromatography-mass spectrometry (GC-MS)-based metabolite profiling [file nph0201-0476-sd1.docx]

**Supporting Information Tables S1–S3 and Figs S1 & S2**

**Table S1** Sequence of primers used in this work

| Gene | Forward Primer | Reverse Primer |
| --- | --- | --- |
| *UGD1* | AGACTCCAGCGATTGATG | GAGACGACATTCACTTGC |
| *UGD2* | AACAACAGTGAAACAAGTGAC | TGAAACCAATCTCCCTTAGC |
| *UGD3* | TGAAGCAAGTCTCAGTCG | GTAAACAATAAACCCAATCTCC |
| *UGD4* | TGATGTGTGTAAAGGTCTATTAGG | ACTGTGGTTGGACTCATTGG |
| *18S* | GGTGGTAACGGGTGACGGAGAAT | CGCCGACCGAAGGGACAAGCCGA |
| *Thi2.1* | TTCCAAGGGAAGGTGTATGC | ACATCCCTTGGCACATTGTT |

**Table S2** Expression of *MIOX* genes in syncytia of the *miox* quadruple mutant measured by qPCR at 10 dpi. Transcripts of *MIOX1*, *MIOX4*, and *MIOX5* were severely downregulated and transcripts of *MIOX2* were not detected (nd). Values are means, *n* = 3.

| **Gene** | **Fold change**  (Col-0 vs *miox1/2/4/5)* | **% Expression** |
| --- | --- | --- |
| ***MIOX1*** | -16.33 | 6.1 |
| ***MIOX2*** | nd | nd |
| ***MIOX4*** | -10.7 | 9.2 |
| ***MIOX5*** | -14 | 7.1 |

**Table S3** Comparison of wild type syncytia and *miox1/2/4/5* syncytia by GC-MS based metabolite profiling.

| **Metabolite** | **Fold change (log_2_)** | **Fold change** |
| --- | --- | --- |
| **Glucose-6-phosphate** | 1.01 | 2.8 |
| **Inositol myo phosphate** | 1.46 | 2.75 |
| **Inositol myo** | 2.11 | 4.31 |
| **Galactinol 9 TMS** | 3.59 | 12.1 |

**Fig. S1 Pathways for the synthesis of UDP-Glucuronic acid, galactinol and derivatives.**

**
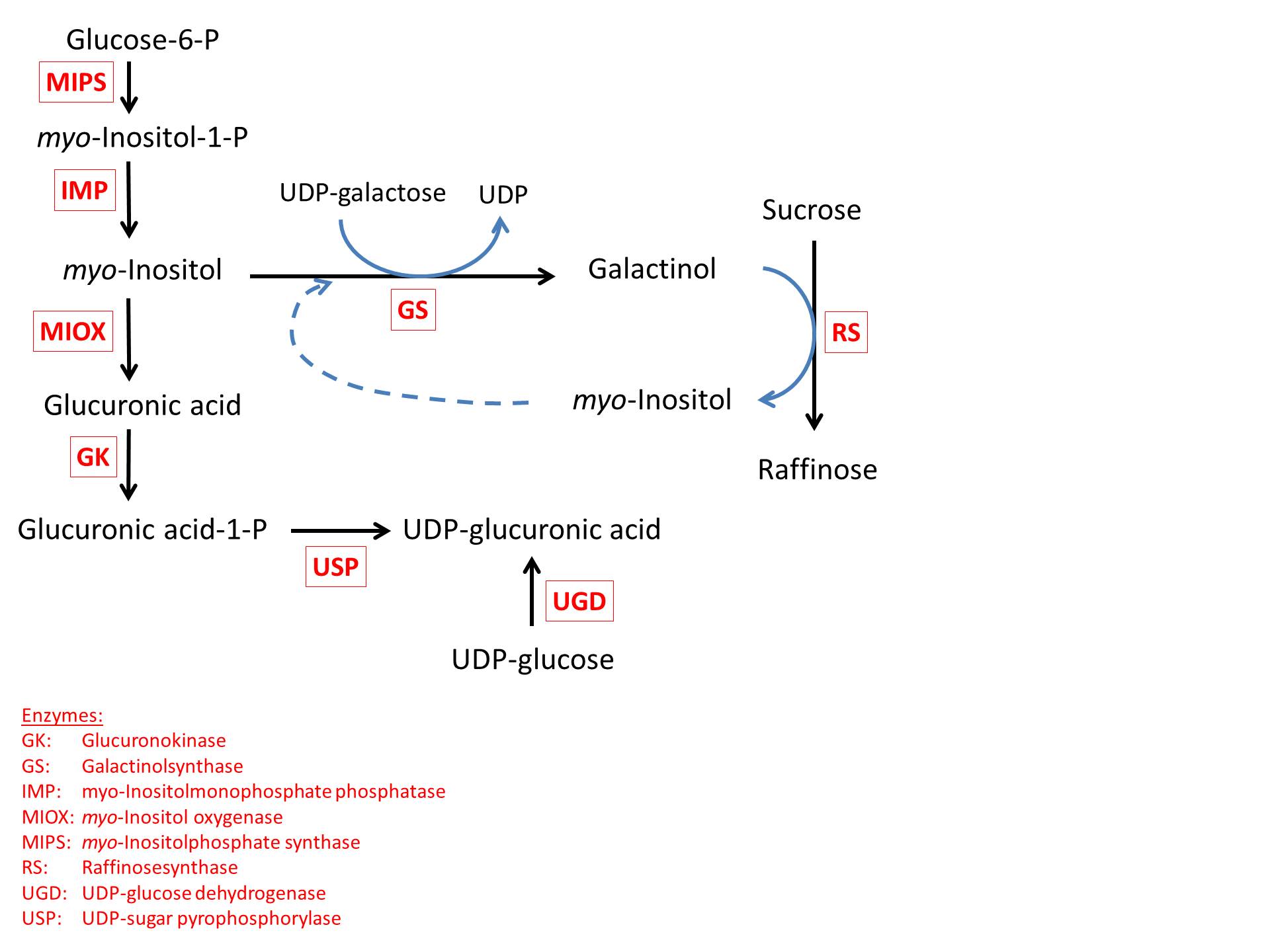
**

**Fig. S2** Light and electron microscope comparison of root and syncytia in Col-0 and miox quadrupule mutants. Nu, nucleus; S, syncytium; X, xylem; Se, sieve elements; N, nematodes.

**
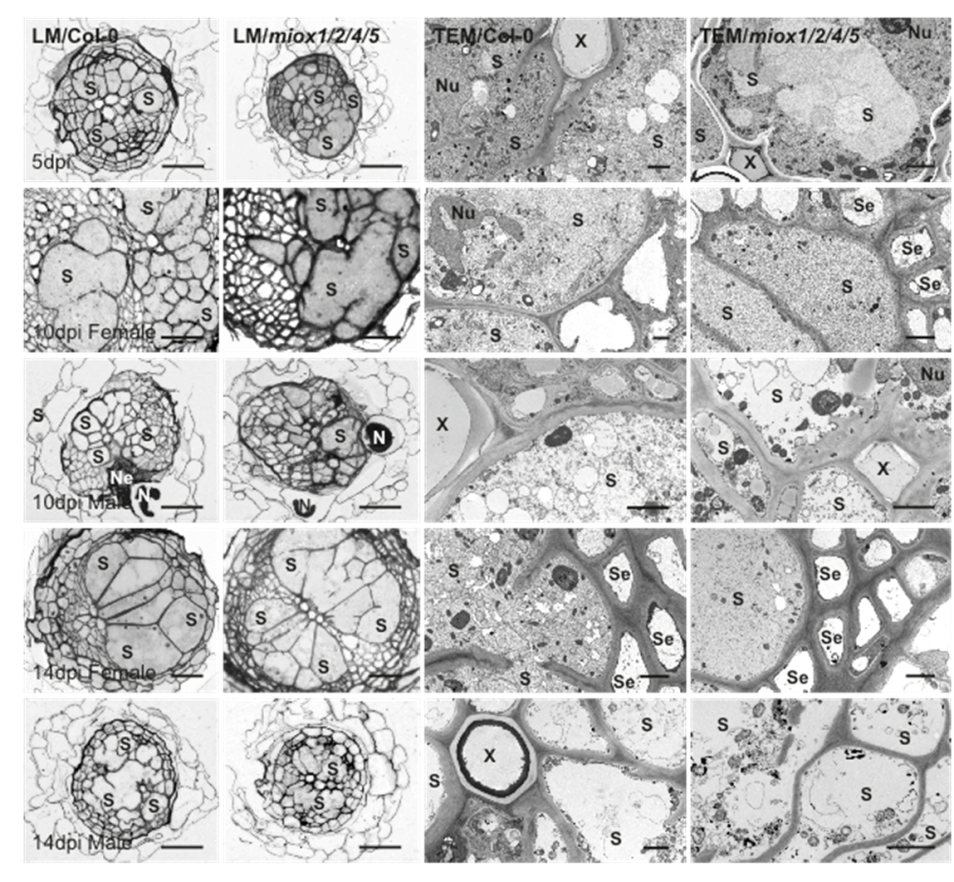
**
